# CCL2 Predicts Survival in Patients with Inoperable Hepatocellular Carcinoma Undergoing Selective Internal Radiotherapy

**DOI:** 10.3390/cancers16162832

**Published:** 2024-08-12

**Authors:** Florian Haag, Severin Gylstorff, Jasmin Bujok, Maciej Pech, Borna Relja

**Affiliations:** 1 Experimental Radiology, Department of Radiology and Nuclear Medicine, Otto-von-Guericke-University, 39120 Magdeburg, Germany; 2 Research Campus STIMULATE, Otto-von-Guericke-University, 39120 Magdeburg, Germany; 3 Department of Radiology and Nuclear Medicine, University Medical Center Mannheim, Heidelberg University, 68167 Mannheim, Germany; 4 Translational and Experimental Trauma Research, Department of Trauma, Hand, Plastic and Reconstructive Surgery, University Ulm, 89081 Ulm, Germany; 5 Department of General Psychiatry, Center for Psychosocial Medicine, Heidelberg University, 69115 Heidelberg, Germany

**Keywords:** HCC, MCP-1, biomarker, diagnosis, prognosis

## Abstract

**Simple Summary:**

Malignant diseases represent a central challenge in modern medicine. Despite improved screening approaches and advanced diagnostics, malignant diseases such as HCC are still diagnosed at non-resectable/advanced stages. In these cases, minimally invasive treatment methods such as SIRT or other radioablation procedures are of particular importance as part of personalized oncological therapy. When choosing the right minimally invasive procedure, it would be useful to be able to use biomarkers as predictors of treatment response. With this background, the aim of this study is to evaluate the usefulness of CCL2 as a predictor of treatment response and survival.

**Abstract:**

Purpose: Hepatocellular carcinoma (HCC) is the largest subgroup of primary liver tumors. Ablative therapies, such as selective internal radiation therapy (SIRT), are used in late stages for patients with unresectable liver metastases and no response to other therapies. CCL2 (C-C motif chemokine ligand 2) is a potent monocyte chemoattractant. It is associated with tumor progression and metastasis. The role of circulating CCL2 as a biomarker in HCC undergoing selective internal radiation therapy remains unclear. Methods: A total of 41 patients (8 female, 33 male) suffering from HCC and undergoing SIRT were enrolled. Pre- and post-therapy changes in circulating CCL2 levels were determined by bead-based immunoassay and compared with clinical laboratory parameters and patient data. Results: A total of 32 patients exhibited survival beyond 60 days. It was observed that levels of CCL2 correlated with scores indicating a higher likelihood of non-survival and with the severity of the disease. Moreover, a significant inverse correlation was discovered between CCL2 levels and the survival of patients over 60 days in relation to counts of leukocytes, granulocytes, monocytes, and C-reactive protein. Conclusions: CCL2 may serve as a potential marker for patient survival after SIRT. The prediction of which HCC patients are likely to benefit from SIRT may be helpful in guiding therapeutic management.

## 1. Introduction

Globally, cancer is a leading cause of death with increasing incidence and mortality rates. In 2020, primary liver cancer was the sixth most diagnosed cancer and the third leading cause of cancer-related death worldwide [1]. Hepatocellular carcinoma (HCC) is an epithelial tumor that develops in the liver and primarily comprises cells that resemble normal hepatocytes [2]. In Europe, alcohol consumption and the resulting liver cirrhosis are the most prevalent causes, with approximately the most HCC cases [3]. Worldwide, the epidemiology of HCC varies between Eastern and Western cohorts; hepatitis B and C are the most critical risk factors for the genesis of HCC. Other risk factors include nonalcoholic steatohepatitis, nonalcoholic fatty liver disease, and exposure to aflatoxin B1 produced by Aspergillus species [4]. There are many stage-dependent and specific approaches to improve HCC survival and treatment choices. According to the Barcelona Clinic Liver Cancer staging system, which is commonly used to stage HCC, 30% of patients are diagnosed at an advanced stage [5,6]. In the majority (>70%) of cases, HCC has progressed so far that surgical resection or liver transplantation can no longer be performed at the time of diagnosis [7,8]. One treatment option for non-resectable HCCs is the transarterial approach of selective internal radiation therapy (SIRT) [7,8]. SIRT uses an arterial catheter to deliver radioactive material (e.g., Yttrium-90) directly into the bloodstream of the tumor to inhibit perfusion, induce necrosis of the tumor tissue, and stimulate the invasion of immune cells [9]. Owing to the heterogeneity in the population within an advanced stage, the benefits and outcomes of SIRT are considered variable [9,10]. The survival of patients with advanced HCC is monitored using a combination of imaging modalities such as computed tomography (CT), contrast-enhanced magnetic resonance imaging (MRI), and ultrasound. However, to a minor extent, these imaging techniques provide immunological information regarding the response to tumor treatment [11]. Local and systemic immune activation is associated with a sustained response to SIRT in HCC patients [11,12]. Chemokine (C-C motif) ligand 2 (CCL2), also known as chemokine Monocyte chemoattractant protein-1 (MCP-1), belongs to this chemokine group. These are small molecules (8–14 kDa) that are secreted, among others, by cells of the immune system [13,14]. As a pro-inflammatory mediator, CCL2 activates monocytes and other immune cells by binding to the C-C chemokine receptor (CCR2). It induces the differentiation of monocytes into macrophages and tissue invasion [15]. CCR2 is also expressed in tumor cells and possesses a pro-cancerogenic function [16]. In vitro studies have shown that CCL2 promotes HCC cell invasion [17]. Furthermore, data from a CCL2 rat model showed that the inhibition of the MCP-1CCL2/CCR2 axes in animals improves the efficacy of transarterial chemoembolization (TACE) [18]. Our own studies with patients after undergoing interstitial HDR brachytherapy of liver metastases correlations were able to show a positive correlation between baseline circulating levels of CCL2 and the tumor volume as well as threshold doses of irradiation damage [19].

Based on this existing evidence, we hypothesized that CCL2 levels in plasma HCC patients undergoing SIRT have prognostic potential and can be identified as markers for patient therapy outcomes.

## 2. Materials and Methods

### 2.1. Ethics

The recent study was conducted in accordance with the Declaration of Helsinki and approved by the institutional ethics committee approval (SWARM RAD298) of the University Hospital of University Magdeburg. Written informed consent was obtained from all the patients.

### 2.2. Study Settings and Population

A total of 41 patients (female, n = 8; male, n = 33) suffering from HCC who underwent SIRT were included in the study.

Inclusion criteria were: (I) HCC, (II) indication for SIRT, (III) chemotherapy and cortisone therapy paused for a minimum of two weeks before admission, and (IV) >18 years of age.

Exclusion criteria were (I) live expectancy <3 months, (II) hepatic tumor load >70%, (III) chronic infections (HIV and Hepatitis Virus), (IV) pronounced ascites, (V) contraindications for angiography, MRI contrast medium (Gd-EOB-DTPA), X-ray contrast medium, MRI, and CT, (VI) severe cardiovascular diseases (NYHA III/IV), (VII) thrombotic or embolic events in the last six months (stroke/transient ischemic attack), (VIII) immunosuppression (e.g., after transplantation) or human immunodeficiency virus, especially cortisone long-term therapy; and (IX) autoimmune diseases or chronic inflammatory bowel diseases.

### 2.3. Technique of 90Y-Radioembolization

**Figure 1:**
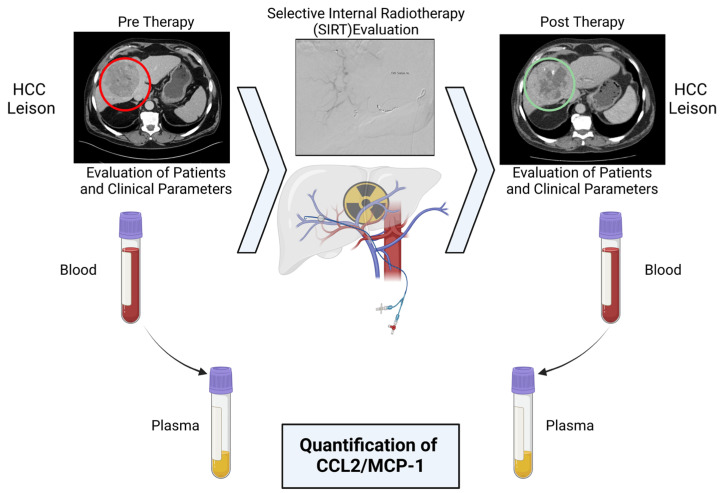
Illustration of elective study design of CCL2 before and after internal radiation therapy (SIRT) (created with BioRender.com). (**Top**) CT scan of a patient with hepatocellular carcinoma (HCC) lesion before and after therapy. The tumor shows typical features such as early contrast enhancement, combined with wash-out in the evaluation phase at the application of 99mTc-MAA via an arterial catheter after isolation of arterial hepatic blood supply. (**Bottom**) CC chemokine ligand 2 (CCL2) and blood, as well as clinical parameters and standard laboratory parameters, were collected and quantified before (pre-T) and after (post-T) therapy.

A detailed description of SIRT is provided elsewhere [20]. Radioembolization was performed using Yttrium-90 (90Y) resin microspheres (SIR-Spheres^®^, Sirtex Medical, Lane Cove, Australia). Before SIRT, angiography was performed to identify the hepatic arterial tree, and arterial feeders. The hepatic arterial blood supply was isolated by coiling additional feeders. In the next step, 99mTc-MAA (150 MBq, 99mTc-LyoMAA, Covidien, Neustadt/Donau, Germany) was applied to the hepatic artery, and a gamma camera (E.CAM 180, Siemens, Erlangen, Germany) was used to detect the extent of hepatopulmonary shunting. A single-photon emission computed tomography (SPECT) scan of the upper abdomen was performed in order to detect extrahepatic non-target seeding of 99mTc-MAA. The body surface area (BSA) method was used to calculate the activity of 90Y resin microspheres [21]. Two weeks after this procedure, 90Y resin microspheres were delivered selectively into the hepatic arteries via a temporary transfemoral catheter (Figure 1).

### 2.4. Data Acquisition and Blood Sampling

Blood samples were collected one day before (pre-T) and two days after (post-T) SIRT. Ethylenediaminetetraacetic acid (EDTA) tubes (Becton Vacutainer; Becton Dickinson Diagnostics, Aalst, Belgium) were used to complete the blood count and plasma collection. Serum collection was performed using clot activator tubes (BD Vacutainer SST™ II Advance Tubes; Becton Dickinson Diagnostics, Aalst, Belgium). Citrate tubes (Becton Vacutainer; Becton Dickinson Diagnostics, Aalst, Belgium) were used for determining coagulation parameters. Blood, plasma, citrate, and serum were directly transferred for clinical routine analysis and analyzed according to DIN EN ISO 15189:2014 and DIN EN ISO/IEC 17025:2018 by the Institute for Clinical Chemistry and Pathobiochemistry and University Hospital Magdeburg [22]. The clinical routine included a complete blood count (WBC) (leukocytes, erythrocytes, platelets, neutrophils, immature granulocytes, eosinophils, basophils, lymphocytes, and monocytes). Serum, citrate, and plasma samples were analyzed for coagulation parameters (quick test, international normalized ratio (INR), partial thromboplastin time (PTT), and thrombin time), clinical parameters (creatinine, urea, uric acid, bilirubin, albumin, alanine aminotransferase (ALT), aspartate aminotransferase (AST), alkaline phosphatase (ALP), and gamma-glutamyl transferase (GGT)) and C-reactive protein (CRP). See the experimental design illustrated in Figure 1.

### 2.5. Quantification of CCL2 by LEGENDPlex™

CCL2 levels in co-isolated plasma samples from patients before and after SIRT liver treatment were measured simultaneously using a multiplex bead-based immunoassay (LEGENDPlexTM Human Neuroinflammation Panel 1, Cat. No. 740795, Biolegend, San Diego, CA, USA). EDTA-treated blood was centrifuged for 15 min at 1500× *g* and was stored at −80 °C until further analysis. The obtained plasma was diluted 1:2 following the manufacturer’s recommendations. Data were collected from patients by flow cytometry in a FACS Celesta™ Cell Analyzer (BD Biosciences, Franklin Lakes, NJ, USA), and the concentration of each factor was calculated using five-parameter logarithmic curves and compared with the Representative Standard Curve using and within the supplier’s software [23].

### 2.6. Statistics

The Plasma CCL2 concentrations and the patient’s data were measured pre- and post-SIRT and analyzed using GraphPad Prism 6.0 software (GraphPad Software Inc., San Diego, CA, USA). All data were tested for normality using the Kolmogorov–Smirnov test. Data are presented as the mean ± standard error of the mean or as otherwise indicated. Differences between HCC survivors and non-survivors were determined using the non-parametric Mann–Whitney test and Wilcoxon matched-pairs signed rank test. Correlation analysis was performed using Spearman’s correlation coefficient and Spearman’s r-test. Statistical significance was set at *p* < 0.05.

## 3. Results

### 3.1. Characteristics of the Study Cohort

In total, 41 patients (8 female and 33 male) undergoing SIRT were included. The imaging, biological, and clinical parameters of HCC patients were collected before and after SIRT and correlated with a 60-day survival (hereafter referred to as the HCC cohort). The baseline patient characteristics of this cohort (Table 1 and Table 2) were summarized. A total of 32 of the 41 patients survived longer than 60 days after therapy, and 9 died. The median age of HCC cohort survivors was 69 [range 63.25–78.75], and the median age of non-survivors was 70 [range 62.00–79.50]. The survivors showed a median total liver volume of 1950 cm3 [range 1484–2482] and a tumor fraction of 5.8%, while non-survivors showed a median liver volume of 2790 cm^3^ [range 1860–3332] and a tumor fraction of 27.40% (Table 1).

### 3.2. Clinical Parameters

**Figure 2:**
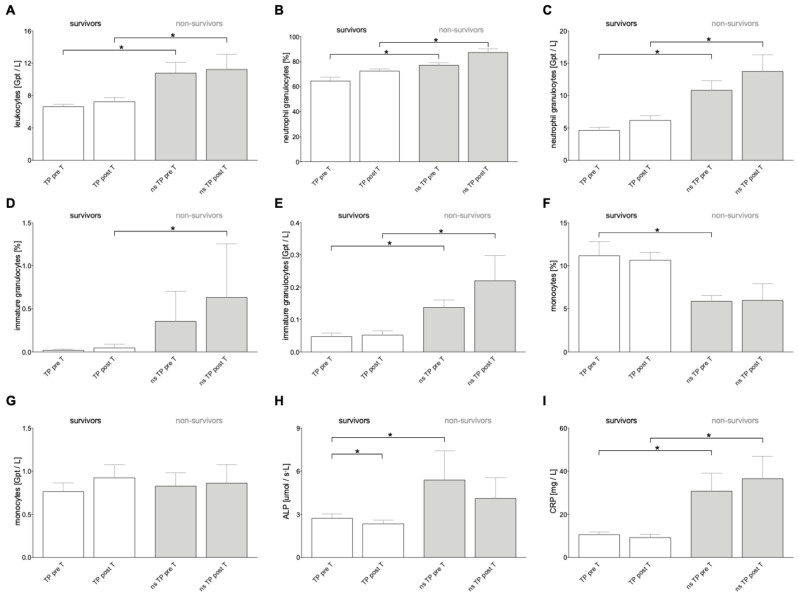
Immune cell distribution, levels of Alkaline phosphatase (ALP), and C-reactive protein (CRP) depending on tumor patients’ (TP) survival status before and after selective internal radiotherapy (pre-T and post-T). Counts of leukocytes in absolute cell numbers [Gpt/L] (**A**), neutrophil granulocytes in percentages [%] (**B**), absolute neutrophil numbers [Gpt/L] (**C**), immature granulocytes in % (**D**), absolute immature granulocytes numbers (**E**), monocytes in % (**F**), monocytes as absolute cell numbers (**G**), levels of ALP (**H**), and CRP (**I**) in survivors and non-survivors (ns) pre-T and post-T are shown. Data are presented as mean ± standard error of the mean. *: *p* < 0.05 vs. indicated group.

It was shown that prior to treatment, Alkaline phosphate (ALP) and C-reactive protein (CRP) were significantly elevated in the non-survival group compared to the survival group (*p* = 0.0352 and *p* = 0.0341, respectively, Table 1 and Figure 2). Following post-therapy, significant differences in CRP levels and aspartate aminotransferase (AST) levels between surviving and non-surviving patients were observed (CRP: *p* = 0.0094 and AST: *p* = 0.0070, respectively, Table 1 and Figure 2). In the survival group, urea, albumin, gamma-glutamyl transferase (GGT), and insulin growth factor (IGF) levels were significantly decreased compared to pre-treatment levels (urea: *p* = 0.0002, albumin: *p* < 0.0001, GGT: *p* < 0.0001, and IGF: *p* = 0.0162, Table 1). Significant changes were detected in creatinine, bilirubin, albumin, AST, and thrombin time levels in the non-survivor group after therapy (pre-T vs. post-T) (creatinine: *p* = 0.0273, bilirubin: *p* = 0.0117, albumin: *p* = 0.0078, AST: *p* = 0.0234, and thrombin time: *p* = 0.0156, Table 1).

### 3.3. Cell Count Pre- and Post-SIRT

In addition to liver parameters, the total number of leukocytes, lymphocytes, granulocytes, monocytes, erythrocytes, and thrombocytes was recorded as part of the study (Table 2 and Figure 2). The cell counts were compared and analyzed pre-T versus post-T and between the survivor group and the non-survivor group (Table 2), as well as within the survivor and non-survivor groups (Table 2). Before therapy, the survivor and non-survivor groups showed significant differences in the levels of leukocytes, neutrophil granulocytes, immature granulocytes, basophil granulocytes, and monocytes (leukocytes: *p* = 0.0018, neutrophils granulocytes: *p* = 0.0008, basophil granulocytes: *p* = 0.0059, and monocytes: *p* = 0.0109, Figure 2).

After treatment completion, significant differences were observed in the levels of leukocytes, neutrophil granulocytes, immature granulocytes, eosinophil granulocytes, and lymphocytes between surviving and non-surviving patients (leukocytes: *p* = 0.0313, neutrophil granulocytes: *p* = 0.0037, immature granulocytes: *p* = 0.0073, eosinophil granulocytes: *p* = 0.0462, and lymphocytes: *p* = 0.0103, Table 2 and Figure 2). In the surviving group, there were significant changes between the pre-T and post-T levels of erythrocytes, thrombocytes, and lymphocytes (erythrocytes: *p* = 0.0050, thrombocytes: *p* = 0.0012, and lymphocytes: *p* = 0.0312, Table 2), while the non-surviving group showed a significant difference in thrombocytes (*p* = 0.0391, Table 2).

### 3.4. CCL2 Levels in Patients Receiving SIRT

**Figure 3:**
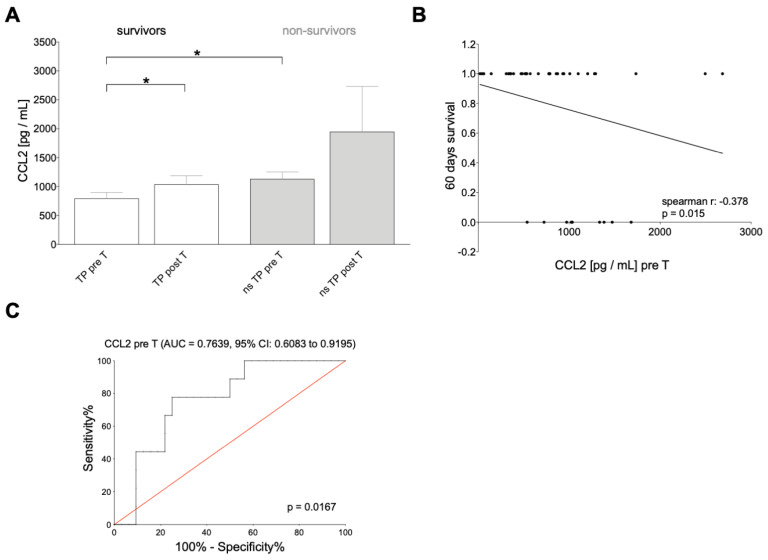
CC chemokine ligand 2 (CCL2) is associated with patient survival status, pre- and post-selective internal radiotherapy. (**A**) Levels of CCL2 in tumor patient (TP) survivors vs. non-survivors (ns) before (pre-T) and after therapy (post-T). (**B**) Negative correlation between CCL2 levels before therapy and 60 days survival. (**C**) Sensitivity and specificity assessment of the prognostic performance of pre-therapy CCL2 levels in the prediction of 60 days survival by receiver operating curve analysis. Data are presented as mean ± standard error of the mean. *: *p* <0.05 vs. indicated group.

In addition to blood levels, patients’ CCL2 levels were measured. These levels were compared with the 60-day survival levels. CCL2 blood levels distinguished significantly pre-T and post-T in survivors and non-survivors. CCL2 levels were significantly higher in pre-T non-survivors than in survivors (Figure 3). CCL2 levels were correlated and showed a significant negative correlation (r = −0.378, *p* = 0.015) with the patient’s 60-day survival (Figure 3). The diagnostic performance of pretreatment CCL2 levels in predicting the 60-day survival by ROC analysis showed a relatively moderate predictive capacity for a response to SIRT with an area under the curve of 0.7639 (CI: 0.6083–0.9195) (Figure 3). At a plasma CCL2 level of 957.1 pg/mL, a sensitivity of 77.78% and a specificity of 75% were observed, resulting in a likelihood ratio of 3.111.

### 3.5. Treatment Response Associated with CCL2 and 60-Day Survival

The patients showed several correlations between treatment parameters like tumor size, tumor fraction, administered activity dose, and measured levels of leukocytes and ALP, CRP, as well as CLL2. Positive significant correlations were observed between tumor size and pre-T leukocytes, neutrophil granulocytes, immature granulocytes, and CRP levels. The tumor fraction positively correlated with pre-T with ALP. The administered activity dose was positively correlated with post-T leukocyte counts. Pre-T Levels of leukocytes showed a significantly positive correlation with neutrophil granulocytes pre- and post-T, immature granulocytes pre- and post-T as ALP post-T, and CRP pre-T and post-T. A negative correlation was observed between pre-T and pre-T monocyte levels. Positive correlations were also observed between pre-T CCL2 and leukocytes (pre- and post-T), immature granulocytes (pre- and post-T), CRP (pre-T), and CCL2 (post-T). Significant negative correlations with the 60-day survival showed for leukocytes (pre-T and post-T), neutrophil granulocytes (pre- and post-T), immature granulocytes (pre- and post-T), ALP (pre-T), CRP (pre-T and post-T), and pre-T CCL2 (Figure 3). Monocytes (pre-T) showed a positive correlation with a 60-day survival (Table 3).

## 4. Discussion

Selective internal radiation therapy is a critical component of cancer treatment. It can be used alone or in combination with other therapies. The correct method and approach to treatment often determine how a therapist perceives the treatment situation and predicts outcome and progression. Predictive independent inflammatory biomarkers may play a central role in the assessment of image-guided therapies and the result of these therapies.

Our research suggests that elevated plasma levels of CCL2 measured before (pre-T) treatment in HCC patients are negatively correlated with survival of the SIRT. This observation is consistent with the evidence presented in numerous preceding studies, demonstrating that CCL2 is associated with adverse prognoses across a diverse spectrum of neoplastic disorders [24,25]. In human liver cancers, among others, Xiaoguang Li et al. showed that CCL2 is an independent prognostic factor that is highly expressed, and its concentration negatively correlates with patient survival [26]. Moreover, blocking the CCL2/CCR2 pathway prevents the recruitment and infiltration of inflammatory monocytes and the M2 polarization of tumor-associated macrophages (TAMs). This reverses the immunosuppressive state within the tumor microenvironment and activates an anti-tumor response by CD8+ T cells [26,27]. In a broader context, our study also showed a correlation between CCL2 levels pre-T and the number of measured leukocytes and immature granulocytes, as well as independent pro-inflammatory markers such as CRP. Matching this, we demonstrated that the number of leucocytes, granulocytes, and monocytes also correlated negatively with 60 days of survival. However, although CCL2 should stimulate the differentiation of monocytes into macrophages and their invasion into the tissue [15], we do not see any significant differences in the absolute number of monocytes in the clinical blood counts. One possible explanation for this could be that CCL2, in addition to the differentiation and invasion of monocytes, also affects the recruitment of new monocytes, as has already been shown in other studies [28], and we, therefore, cannot demonstrate any net changes in the total cell count. For a better understanding, further studies in which cell activity and subsets are also specifically investigated would be useful here. Unfortunately, the present results do not allow a description of the cells from which circulating CCL2 is derived. However, it can be assumed that CCL2 originates from immune cells, tumor cells, and endothelial progenitor cells. Specifically, Tuo Deng et al. have demonstrated in HCC that endothelial progenitor cells release a higher quantity of CCL2, which stimulates the upregulation of CD36 and thus supports the pro-metastatic microenvironment [29]. This could explain why there is no significant correlation between the tumor volume or the tumor fraction of the liver, and CCL2 could be proven. Despite the described factors, the influence of CCL2 on the tumor environment should also be mentioned. It has already been shown that cancer-associated fibroblasts, which belong to the tumor microenvironment, secrete CCL2 and induce functional reprogramming of monocytes to immunoinhibitory myeloid-derived suppressor cells [30]. Besides other cell types, these cells are associated with radiation resistance [31].

Given this background, it seems reasonable to hypothesize that affected patients might benefit from a CLL2/CCR2 axis blockade [18]. Tian et al. have already tested this therapeutic approach in an animal model. They showed prolonged survival in rats suffering HCC and receiving TACE in combination with a selective CCR2 antagonist [18,32]. Fitting to the recent study, their work highlights the therapeutic potential in CCL2/CCR2. It raises the assumption that patients undergoing SIRT, which is also a transarterial ablation of cancerous tissue, would benefit from this approach [32]. Thereby, A. Wiesemann et al. showed in mice lungs that inhibiting radiation-induced CCL2 signaling protects the lung from vascular dysfunction and endothelial cell loss [33].

Another important conclusion from the presented results is that CCL2 can be used as a marker for the suitability of patients for SIRT. Although SIRT is well established and considered minimally invasive, it can be tiring for patients who are mostly in a more advanced stage of the disease, especially because of the minimum two-stage procedure. Therefore, it is particularly important to select the patients who are eligible for SIRT carefully. Currently, the evaluation of patient suitability is based on clinical criteria, such as liver function and the patient’s performance status, according to the Eastern Cooperative Oncology Group (ECOG) [34]. Furthermore, previous work identified elevated baseline transaminases (ALT/AST) and an ECOG performance status ≥1 as prognostic factors for breast cancer patients receiving SIRT [35]. Also, high levels of bilirubin and transaminases were shown to be risk factors for HCC patients undergoing SIRT for experiencing a decline in liver function [36]. Taken together, these data underline that the patients have to be evaluated accurately. In this context, the serum level of CCL2 is a promising marker for 60 days of survival after therapy and can be helpful in the therapy decision. Other parameters that were associated with the 60-day survival that can be addressed were the count of leucocytes, neutrophils, and immature granulocytes, as well as ALP and CRP.

The main limitation of the study is the relatively small patient cohort. It has to be investigated if CCL2 is also predictive of a worsened outcome in HCC patients receiving another therapy or patients with other tumors affecting the liver. An additional limitation may be that we are unable to definitively rule out the alcohol intake of patients prior to treatment. As previous studies have shown alcohol consumption can raise CCL2 blood levels [37,38], this cannot be ruled out as a distractor. Also, while our study provides valuable insights, the lack of comprehensive metabolic data and the potential for confounding metabolic syndrome and related conditions should be taken into account when interpreting the results. The presence of metabolic syndrome or diabetes in the comparison groups was not systematically assessed, which could introduce bias given that CCL2 is implicated in these conditions. Additionally, BMI data for all patients were not consistently available. Cachexia in advanced tumor stages can indicate prior obesity, suggesting that baseline BMI measurements and clinical data on weight loss would be valuable. The lack of comprehensive BMI data limits our ability to fully control for obesity-related inflammation. CCR2′s role in adipose tissue inflammation within the context of obesity and metabolic diseases further complicates our findings. While extensively studied in other inflammatory conditions, CCR2′s relevance to metabolic syndrome is debated, necessitating careful interpretation of our results. Due to these gaps, the potential confounding effects of metabolic syndrome and related conditions cannot be entirely ruled out. Future research should include thorough assessments of metabolic health and related clinical parameters to provide more robust findings.

## 5. Conclusions

In summary, the present work demonstrates that elevated levels of CCL2 in the serum of patients with unresectable HCC are associated with reduced survival after SIRT. This suggests that CCL2 may be a predictor of survival after therapy and may be helpful in making treatment decisions. Similarly, the results suggest that it needs to be investigated whether patients would benefit from a blockade of the CCL2/CCR2 axis.

## Figures and Tables

**Table 1:** Patient cohort characteristics and clinical parameters: Median value with 75% percentile is given. Median value with 75% percentile is given.

Variables	Pre-T		Post-T		*p* Value < 0.05
	Survivors (n = 32)	Non-Survivors (n = 9)	Survivors (n = 32)	Non-Survivors (n = 9)	
Age [median (range)]	69.00 (63.25–78.75)	70.00 (62.00–79.50)	69.00 (62.50–79.00)	70.00 (62.00–79.50)	
Gender (female, n)	7	1			
Total liver volume (cm^3^) [median (range)]	1925 (1484–2482)	2790 (1860–3332)			
Tumor volume (cm^3^) [median (range)]	174.30 (70.17–529.00)	690.00 (133.10–1836.00)			
Tumor fraction (%) [median (range)]	5.80 (3.81–22.40)	27.40 (7.00–42.68)			
Administered activity dose (MBq) [median (range)]	1190 (1003–1392)	1438 (1010–1579)			
Creatinine (umol/L) [median (range)]	83.50 (66.00–100.50)	72.00 (60.50–88.50)	82.00 (70.00–105.00)	87.00 (73.00–108.00)	d
Urea (mmol/L) [median (range)]	5.15 (3.36–7.95)	4.80 (4.10–7.20)	6.60 (4.40–8.80)	8.10 (5.25–9.90)	c
Uric acid (umol/L) [median (range)]	373.00 (284.00–441.00)	317.00 (249.00–451.00)	350.00 (271.00–393.00)	395.00 (251.50–530.00)	
Bilirubin (umol/L) [median (range)]	10.20 (8.48–15.65)	12.40 (8.00–15.85)	14.30 (8.90–20.80)	17.80 (9.90–23.55)	d
Albumin (g/L) [median (range)]	39.40 (36.63–42.93)	39.40 (34.00–42.15)	37.10 (33.50–40.60)	36.60 (29.70–39.30)	c, d
Alanine aminotransferase (umol/s·L) [median (range)]	0.66 (0.43–1.10)	0.79 (0.52–1.07)	0.71 (0.44–0.99)	0.72 (0.55–2.16)	
Aspartate aminotransferase (umol/s·L) [median (range)]	0.88 (0.66–1.30)	1.23 (0.65–2.11)	0.73 (0.54–1.35)	2.10 (1.06–8.10)	b, d
Alkaline phosphatase (umol/s·L) [median (range)]	2.19 (1.52–3.18)	3.64 (2.20–5.22)	1.90 (1.35–2.98)	3.10 (1.69–4.03)	a, c
Gamma-glutamyl transferase (umol/s·L) [median (range)]	2.85 (1.66–6.87)	4.84 (2.03–20.50)	2.51 (1.64–6.38)	3.51 (1.73–23.55)	c
Quick value (%) [median (range)]	87.00 (76.00–94.00)	86.00 (78.00–98.00)	81.00 (73.00–96.50)	75.00 (57.50–92.50)	
INR [median (range)]	1.080 (1.030–1.160)	1.080 (1.010–1.140)	1.120 (1.020–1.175)	1.170 (1.040–1.360)	
PTT (sec.) [median (range)]	29.40 (27.10–34.30)	30.50 (25.90–30.85)	29.20 (27.05–31.85)	30.00 (26.80–33.65)	
Thrombin time (sec.) [median (range)]	16.80 (16.28–17.85)	16.45 (15.65–17.98)	17.70 (16.85–18.60)	18.45 (16.75–19.38)	c, d
C-reactive protein (mg/L) [median (range)]	12.30 (4.84–14.10)	21.30 (8.80–58.80)	6.40 (2.90–11.10)	18.70 (8.70–69.25)	a, b
HGH (ng/mL) [median (range)]	0.586 (0.221–1.320)	0.975 (0.317–3.563)	0.621 (0.342–1.295)	1.91 (0.59–4.37)	
IGF (ng/mL) [median (range)]	71.15 (58.13–109.00)	65.75 (41.55–115.80)	61.20 (50.15–92.55)	74.30 (39.45–111.80)	c

Significance dynamics: (a) pre-T in the survivor group versus the non-survivor group; (b) post-T in the survivor group versus the non-survivor group; (c) survivors pre-T versus post-T; (d) non-survivor pre-T versus post-T.

**Table 2:** Blood counts before (pre-T) and after (post-T) receiving selective internal radiotherapy. Median value with 75% percentile is given.

Variables	Pre-T		Post-T		*p* Value < 0.05
	Survivors (n = 32)	Non-Survivors (n = 9)	Survivors (n = 32)	Non-Survivors (n = 9)	
Leukocytes (Gpt/L) [median (range)]	6.47 (5.31–7.80)	11.20 (6.94–12.85)	6.81 (5.08–8.63)	10.90 (6.81–14.45)	a, b
Erythrocytes (Tpt/L) [median (range)]	4.30 (3.63–4.64)	4.43 (4.03–4.92)	4.29 (3.73–4.54)	4.48 (4.06–4.70)	c
Thrombocytes (Gpt/L) [median (range)]	164.50 (115.80–240.80)	244.00 (159.50–467.50)	146.00 (99.00–219.00)	184.00 (97.50–354.00)	c, d
Neutrophil granulocytes (%) [median (range)]	67.00 (56.95–73.00)	76.30 (73.85–81.45)	73.00 (67.40–75.00)	87.15 (82.18–92.80)	a, b
Neutrophil granulocytes (Gpt/L) [median (range)]	4.88 (3.05–5.77)	9.85 (8.58–13.95)	5.84 (3.76–7.800)	12.83 (9.24–19.08)	a, b
Immature granulocytes (%) [median (range)]	0.00 (0.00–0.01)	0.01 (0.00–1.05)	0.00 (0.00–0.00)	0.01 (0.00–1.89)	b
Immature granulocytes (Gpt/L) [median (range)]	0.04 (0.02–0.06)	0.16 (0.09–0.17)	0.04 (0.02–0.06)	0.21 (0.08–0.38)	a, b
Eosinophil granulocytes (%) [median (range)]	2.00 (1.10–2.75)	1.25 (0.30–3.25)	1.60 (0.00–2.00)	0.10 (0.00–0.28)	
Eosinophil granulocytes (Gpt/L) [median (range)]	0.13 (0.09–0.21)	0.16 (0.07–0.57)	0.08 (0.05–0.15)	0.03 (0.00–0.06)	b
Basophil granulocytes (%) [median (range)]	1.00 (0.75–1.00)	0.40 (0.08–0.58)	0.80 (0.00–1.00)	0.15 (0.25–0.20)	a
Basophil granulocytes (Gpt/L) [median (range)]	0.05 (0.04–0.06)	0.06 (0.04–0.08)	0.05 (0.03–0.06)	0.03 (0.02–0.04)	
Lymphocytes (%) [median (range)]	21.00 (16.00–25.50)	15.30 (8.20–19.63)	14.00 (11.00–19.20)	4.65 (2.08–10.60)	b, c
Lymphocytes (Gpt/L) [median (range)]	1.43 (1.16–1.72)	1.83 (1.35–2.46)	1.07 (0.73–1.49)	0.68 (0.39–1.26)	
Monocytes (%) [median (range)]	9.99 (8.35–12.95)	5.75 (4.63–7.25)	10.00 (9.00–12.00)	5.15 (2.90–9.88)	a
Monocytes (Gpt/L) [median (range)]	0.68 (0.52–0.99)	0.81 (0.54–1.14)	0.79 (0.54–1.400)	0.93 (0.44–1.23)	

Significance dynamics: (a) pre-T in the survivor group versus the non-survivor group; (b) post-T in the survivor group versus the non-survivor group; (c) survivors pre-T versus post-T; (d) non-survivor pre-T versus post-T.

**Table 3:** Correlations between selective internal radiotherapy parameters and the measured levels of leukocytes and Alkaline phosphatase, C-reactive protein, and CC chemokine ligand 2 (CLL2). The table shows the Spearman correlation between the measured parameters before (pre-T) and after therapy (post-T). A *p*-value <0.05 was considered as significant. Also, the Spearman correlation coefficient r is given.

Parameters		Tumor Size (cm^3^)		Tumor Fraction (%)		Administered Activity Dose (MBq)		Leukocytes Pre-T (Gpt/L)		CCL2 Pre-T (pg/mL)		Interval Until Death (Days)		60 Days Survival	
	Time Point	*r*	*p* Value	*r*	*p* Value	*r*	*p* Value	*r*	*p* Value	*r*	*p* Value	*r*	*p* Value	*r*	*p* Value
Leukocytes (Gpt/L)	pre-T	0.361	0.022	0.357	0.062	0.508	<0.001			0.396	0.010	0.326	0.201	−0.473	0.002
	post-T	0.123	0.457	0.127	0.521	0.451	0.004	0.654	<0.001	0.445	0.004	0.064	0.808	−0.342	0.031
Neutrophil granulocytes (%)	pre-T	0.529	0.035	0.400	0.286	0.326	0.202	0.744	0.001	0.314	0.220	−0.214	0.645	−0.538	0.026
	post-T	−0.075	0.790	−0.067	0.865	0.013	0.965	0.330	0.229	0.479	0.071	−0.251	0.515	−0.717	0.003
Neutrophil granulocytes (Gpt/L)	pre-T	0.744	0.001	0.583	0.099	0.549	0.022	0.958	<0.001	0.429	0.086	0.143	0.760	−0.736	0.001
	post-T	−0.146	0.603	−0.017	0.966	0.093	0.742	0.509	0.052	0.563	0.074	−0.033	0.932	−0.698	0.004
Immature granulocytes (%)	pre-T	0.212	0.431	0.440	0.235	−0.154	0.557	0.137	0.599	−0.142	0.587	0.612	0.144	−0.468	0.058
	post-T	−0.152	0.589	−0.266	0.489	−0.160	0.568	0.154	0.584	0.081	0.774	−0.488	0.183	−0.522	0.046
Immature granulocytes (Gpt/L)	pre-T	0.534	0.033	0.599	0.088	0.531	0.028	0.758	<0.001	0.587	0.013	0.709	0.074	−0.658	0.004
	post-T	−0.022	0.939	0.294	0.422	0.164	0.569	0.590	0.021	0.563	0.032	0.119	0.761	−0.669	0.006
Monocytes (%)	pre-T	−0.313	0.238	−0.319	0.402	−0.151	0.564	−0.578	0.015	−0.083	0.752	0.414	0.355	0.613	0.009
	post-T	−0.475	0.074	−0.283	0.460	−0.171	0.541	−0.324	0.240	−0.271	0.328	0.100	0.798	0.489	0.065
Monocytes (Gpt/L)	pre-T	0.271	0.311	0.233	0.546	0.284	0.269	0.244	0.345	0.402	0.110	0.643	0.119	−0.057	0.829
	post-T	−0.238	0.384	0.133	0.732	0.202	0.470	0.331	0.228	0.216	0.439	0.460	0.231	0.035	0.902
Alkaline phosphatase (umol/s·L)	pre-T	0.222	0.170	0.376	0.049	−0.249	0.116	0.199	0.213	0.107	0.505	−0.480	0.051	−0.331	0.034
	post-T	0.275	0.090	0.241	0.216	−0.143	0.378	0.152	0.351	0.164	0.311	−0.463	0.061	−0.239	0.138
C-reactive protein (mg/L)	pre-T	0.361	0.022	0.430	0.022	0.161	0.315	0.391	0.011	0.152	0.344	−0.059	0.823	−0.334	0.033
	post-T	0.187	0.255	0.067	0.734	0.266	0.097	0.358	0.023	0.403	0.010	−0.102	0.698	−0.407	0.009
CCL2 (pg/mL)	pre-T	0.241	0.135	0.191	0.329	0.271	0.087	0.396	0.010			−0.199	0.445	−0.378	0.015
	post-T	0.100	0.540	0.177	0.368	0.065	0.688	0.106	0.511	0.537	<0.001	−0.265	0.305	−0.174	0.276

## Data Availability

The data can be obtained, upon reasonable request, from the corresponding author.
